# Best Practices in Microbial Experimental Evolution: Using Reporters and Long-Read Sequencing to Identify Copy Number Variation in Experimental Evolution

**DOI:** 10.1007/s00239-023-10102-7

**Published:** 2023-04-03

**Authors:** Pieter Spealman, Titir De, Julie N. Chuong, David Gresham

**Affiliations:** 1 Department of Biology, New York University, New York, NY 10003 USA; 2 Center for Genomics and Systems Biology, New York University, New York, NY 10003 USA

**Keywords:** Copy-number variant, Long term experimental evolution, Fluorescent CNV reporter, Long-read sequencing, Genome dynamics

## Abstract

Copy number variants (CNVs), comprising gene amplifications and deletions, are a pervasive class of heritable variation. CNVs play a key role in rapid adaptation in both natural, and experimental, evolution. However, despite the advent of new DNA sequencing technologies, detection and quantification of CNVs in heterogeneous populations has remained challenging. Here, we summarize recent advances in the use of CNV reporters that provide a facile means of quantifying de novo CNVs at a specific locus in the genome, and nanopore sequencing, for resolving the often complex structures of CNVs. We provide guidance for the engineering and analysis of CNV reporters and practical guidelines for single-cell analysis of CNVs using flow cytometry. We summarize recent advances in nanopore sequencing, discuss the utility of this technology, and provide guidance for the bioinformatic analysis of these data to define the molecular structure of CNVs. The combination of reporter systems for tracking and isolating CNV lineages and long-read DNA sequencing for characterizing CNV structures enables unprecedented resolution of the mechanisms by which CNVs are generated and their evolutionary dynamics.

From the earliest studies of the genetic basis of adaptive evolution using microbial experimental evolution, copy number variants (CNVs) have been known to play a central role in rapid adaptive evolution. Foundational studies in nutrient-limited chemostats discovered that amplifications of genes encoding transporters of the limiting nutrient were repeatedly selected in different bacteria including *Escherichia coli* limited for lactose (Horiuchi et al. 1963) and *Salmonella typhimurium* limited for different carbon sources (Sonti and Roth 1989). Subsequent studies of *Saccharomyces cerevisiae* in glucose-, phosphate-, sulfur-, and nitrogen-limited chemostats revealed the generality of this class of adaptive response (Hansche 1975; Brown et al. 1998; Gresham et al. 2008, 2010; Kao and Sherlock 2008; Hong and Gresham 2014; Payen et al. 2014). CNVs have also been frequently observed in other microbial experimental evolution systems including batch culture serial transfer (Hull et al. 2017; Blount et al. 2020), and selection for drug tolerance (Selmecki et al. 2008; Todd and Selmecki 2020; Bergin et al. 2022; Tomanek and Guet 2022). However, the repeatability of CNVs in chemostat evolution experiments makes them an ideal system for studying CNV dynamics (Ziv et al. 2013; Gresham and Dunham 2014; Gresham and Hong 2015; Ziv et al. 2013; Gresham and Hong 2015). As CNVs underlie phenomena ranging from speciation to tumor evolution (Conant and Wolfe 2008; Stratton et al. 2009; Shlien and Malkin 2009; Zuellig and Sweigart 2018), the study of CNV dynamics, and mechanisms of their formation, in experimental evolution is of broad relevance.

In this paper we present two approaches that we have employed to study CNVs in the context of experimental evolution in chemostats. The first method entails the use of a CNV reporter, which we developed to study CNVs at a specific locus of interest. This straightforward method relies on the use of a phenotypic reporter of gene amplifications and deletion at a locus in single cells. Although conceptually simple, this system provides unparalleled resolution of CNV dynamics and practical utility in the identification and isolation of CNV-containing lineages. The power of the CNV reporter system is complemented by the use of long-read DNA sequencing using Oxford Nanopore Technology (ONT) to characterize the complex novel structures that can arise in genomes during experimental evolution. Although the motivation for the development of these methods has been understanding CNVs in the context of experimental evolution in chemostats, this combination of tools has general utility for a variety of approaches to experimental evolution as well as potential applications in other experimental systems.

## Using Reporter Systems to Interrogate Locus-Specific CNV Dynamics

### Introduction

One of the major limitations in studying CNVs in evolving populations is the challenge of identifying alleles at low frequencies in heterogeneous populations. Typical methods to detect CNVs include DNA sequencing, quantitative PCR, Southern blotting, and DNA microarrays. However, these molecular methods are best suited to the analysis of clonal samples and are unreliable for detecting de novo CNVs in heterogeneous populations. Although in principle it is possible to sample populations, isolate clones, and analyze CNVs using these methods, this approach is not practical in terms of cost, effort, and time. By comparison, the estimation of single-nucleotide variants (SNVs) allele frequencies from Illumina short-read sequencing data are routine and widely used to study SNV dynamics in experimental evolution. The inherent limitations of existing approaches for accurately identifying CNVs have hindered studies of the evolutionary dynamics of CNVs.

**Fig. 1:**
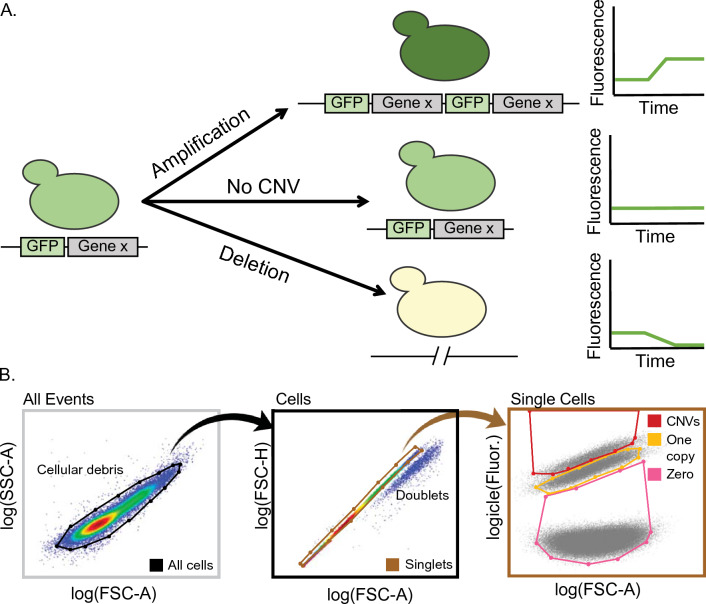
Using CNV reporters to detect de novo CNVs in evolving populations. **A** A CNV reporter consists of a constitutively expressed fluorescent gene (GFP in this example) and drug resistance gene inserted adjacent to, but not fused to, a target gene of interest (Gene x). To track CNVs in real-time during experimental evolution, cell fluorescence is measured using a sample of 100,000 cells from the population on a flow cytometer every 8–16 generations. When the target gene gains or loses copies in the genome, the adjacent reporter gene is amplified or deleted along with it, resulting in a proportional increase or decrease in fluorescence levels, respectively (top and bottom). Fluorescence levels remaining stable over generations indicate that copy number variation has not occurred (middle). **B** Hierarchical gating of flow cytometry data are used for identifying CNV lineages. Left: In samples containing zero-, one-, and two- GFP copy control cells, all flow cytometer events are plotted using forward scatter area (FSC-A) on the *x*-axis and side scatter area (SSC-A) on the *y*-axis. All yeast cells are selected by drawing a gate (black outline), which excludes cellular debris on the basis of smaller size. This defines the Cells gate. Center: Only the events selected by the black Cells gate are carried forward (black arrow), and plotted with FSC-A on the *x*-axis and forward scatter height (FSC-H) on the *y*-axis. Here, we draw a gate (brown outline) only around the cells that lie on the diagonal, which selects for single-cells and excludes any doublets. This defines the Singlets gate. Right: Only the events selected by the brown Singlets gate are carried forward (brown arrow) in our final plot. Plotting the FSC-A on the *x*-axis and the GFP fluorescence channel area on the *y*-axis gives three distributions, corresponding to cells with zero-, one- and two or more-copies of GFP, and consequently, the copy numbers of the gene of interest. Non-overlapping but adjacent gates for each of the three populations are drawn on the basis of engineered control strains containing known numbers of the GFP gene. Pink, orange and red gates correspond to zero-, one-copy- and CNV-containing cells, respectively (Color figure online)

To overcome these challenges, we developed a CNV reporter that enables efficient and real-time tracking of CNV dynamics in evolving populations with single-cell resolution (Lauer et al. 2018). A CNV reporter comprises a constitutively expressed fluorescent gene inserted adjacent to the gene of interest. It directly detects the occurrence of de novo gene amplifications and deletions in individual cells, without requiring molecular analysis (Fig. 1A). Using flow cytometry to quantify fluorescence in individual cells facilitates efficient and rapid CNV allele frequency estimation. Moreover, the reporter system enables efficient isolation of CNV-containing lineages using fluorescence-activated cell sorting (FACS) for further characterization. DNA sequencing of clonal isolates has revealed that CNV reporters detect many different classes of CNVs, including aneuploidies, nonreciprocal translocations, tandem duplications, and complex structural alterations.

### Construction

CNVs often comprise large genomic regions of several kilobases that can include multiple neighboring genes. The principle of a CNV reporter is that when a constitutively expressed fluorescent reporter gene is inserted adjacent to a target gene, CNVs involving the gene of interest can be detected using the expression level of the reporter (Lauer et al. 2018). When the gene of interest is amplified or deleted, the adjacent reporter gene is also amplified or deleted, resulting in an increase or decrease in fluorescence, whereas stable fluorescence levels indicate that copy number at the locus remains invariant. The reporter is integrated at the locus of interest using targeted genome engineering. Therefore, the reporter construct contains both a drug resistance selectable marker and a fluorescent protein gene. As long as insertion of the reporter does not affect the molecular regulation of the target gene, we have found no evidence that CNV formation and selection are affected by the reporter, or that the molecular features of CNVs are affected by the presence of the reporter (Lauer et al. 2018). Although the use of an inducible fluorescent reporter might in principle present advantages, the use of a constitutively expressed protein obviates the need for optimizing additional experimental steps for reproducible expression induction, thereby facilitating rapid and accurate analysis of CNVs.

Constitutive expression of a heterologous gene may be expected to confer negative fitness effects. However, we have confirmed the absence of a fitness defect due to the reporter using pairwise competition assays with isogenic strains that lack the reporter (Lauer et al. 2018). Moreover, maintenance of stable protein fluorescence in one- and two-copy control populations, in which the fluorescent gene is integrated at neutral loci, is consistent with the absence of a detectable fitness cost associated with the CNV reporter.

When developing the reporter system, we initially focused on the general amino acid permease gene, *GAP1*, in the budding yeast *S. cerevisiae*. *GAP1* encodes a high-affinity transporter for amino acids and is highly expressed in nitrogen-deficient conditions (Grenson et al. 1970; Stanbrough and Magasanik 1995) Evolution in the presence of a single nitrogen source has been shown to select for two classes of *GAP1* CNVs: (i) *GAP1* amplification alleles are selected in glutamine and glutamate-limited chemostats, (ii) *GAP1* deletion alleles are selected in urea- and allantoin-limited chemostats (Gresham et al. 2010; Hong and Gresham 2014). Given these prior observations, the *GAP1* locus was ideally suited to testing and demonstrating the versatility of the CNV reporter.

To construct the reporter, we integrated a constitutively expressed (using the *ACT1* promoter) green fluorescent protein (GFP) variant mCitrine (Griesbeck et al. 2001), referred to here as GFP, and kanamycin resistance gene in a region of unique DNA sequence 1118 bases upstream of the *GAP1* start codon. We selected this region so that it is distal to known regulatory regions of *GAP1* and any proximate genes. However, the proximity of the reporter to *GAP1* ensures that it is co-amplified when CNVs are generated at the *GAP1* locus. We use standard genome engineering practices including clonal isolation, molecular confirmation, and backcrossing to confirm correct integration of the reporter. In our experience, the reporter operates as expected and reports on *GAP1* CNVs. In principle, it is possible for *GAP1* CNVs to occur that do not include the reporter; however, in extensive sequence analyses we have not found evidence for this indicating that the reporter system is able to detect all occurrences of *GAP1* CNVs. We have extended the reporter system to other fluorescent genes (mCherry) and loci (*PUT4* and *MEP2*) and observed similar utility as a CNV reporter (unpublished). In constructing different CNV reporters we have placed the construct ~ 1 kb upstream or downstream of the coding sequence. In principle, the CNV reporter should be effective at any locus of interest.

### Copy Number Control Strains

Detecting CNVs using the reporter systems requires the use of three important control strains: (i) a one-copy control containing a single copy of the CNV reporter at a neutral locus (we use the *HO* locus), (ii) a two-copy control containing two copies of the CNV reporter at two neutral loci (we use *HO* and the dubious ORF *YLR123C*), and (iii) a zero-copy control that lacks the CNV reporter. As protein fluorescence is dependent on growth conditions and flow cytometry measurements can be impacted by several experimental variables, the three control strains are propagated in the same selection conditions as the experimental lineages and analyzed in parallel with experimental samples. These control strains serve two functions: (1) they are used to define flow cytometry gates for CNV detection and (2) they provide a measure of CNV reporter stability during the experiment.

We have observed that control populations containing one or two copies of the fluorescent reporter at neutral loci exhibit stable fluorescence for the duration of the evolution experiment (Lauer et al. 2018). To quantify the proportion of cells containing an amplification at the locus of interest, we use the zero-, one- and two-copy control strains to define flow cytometry gates. Propagated control strains are used to define gates for the corresponding time point of the experimental populations to account for variation in experimental procedures and measurements. In practice, we have found that the fluorescent signal in propagated control strains show minimal deviance across the course of the experiment making it appropriate to also use initial flow cytometry measurements of control strains to define gates.

We note that as the control populations are maintained in a selective condition throughout the evolution experiment, they also undergo adaptive evolution. Indeed, sequence analysis of clones isolated from these control populations has identified CNVs at multiple loci. However, the fluorescent signal from the reporter integrated at neutral loci remains stable in these strains consistent with the specificity of the reporter for CNVs at the locus of interest.

### Real-Time Monitoring of CNVs

Flow cytometry analysis is used to monitor CNVs in real-time using the reporter system. We sample each evolving population every few generations (typically every 8–10 generations, which corresponds to 2 days in glutamine-limited chemostats) and measure the fluorescence of a sample from the population. Due to the large population sizes used in our experiments, we routinely measure 10,000–100,000 cells per sample to minimize sampling bias. We analyze cells on the flow cytometer immediately after sample collection, providing real-time tracking of CNVs. In addition, every 16–20 generations, we freeze samples down as cell pellets for subsequent analysis. By the end of a long-term experimental evolution, we typically have ~ 25 timepoints across ~ 250 generations. This timescale is sufficient for observing de novo CNVs under conditions of strong selection (Lauer et al. 2018).

Protein fluorescence increases with cell size and thus cell size must be accounted for to effectively use a CNV reporter. Therefore, we normalize the fluorescent signal for each cell by the cell size as estimated using the forward scatter measurement on a flow cytometer (FSC), resulting in a protein concentration estimation. By engineering strains with different copy numbers of the reporter we have found that the concentration of fluorescent protein is proportional to the ploidy normalized copy number of the fluorescent protein gene, i.e., one-copy in a haploid results in a signal equivalent to two copies in a diploid, and two copies in a haploid results in a signal similar to four copies in a diploid (Lauer et al. 2018). Thus, the cell size–normalized fluorescent signal, or concentration, is an accurate measure of the number of copies of the fluorescent gene in single-cells. The reporter can be used to estimate higher copy numbers as we have found that the normalized fluorescent signal correlates well with copy number estimated from whole genome sequencing data (Lauer et al. 2018).

### Flow Cytometry Analysis for Studying Population-Level CNV Dynamics

We have used a variety of flow cytometers and fluorescent-activated cell sorting (FACS) machines to analyze CNV dynamics using the CNV reporter. Although each machine typically has some sort of analysis software, our standard practice is to export results from these machines as .fcs files and undertake our own analysis using R. This provides greater flexibility and ensures reproducible computational analysis. We use the R package *cytoexploreR* for analysis of flow cytometry data (Hammill 2021). We briefly outline our analysis procedures below and refer the reader to a vignette that provides example code for our analysis (https://greshamlab.bio.nyu.edu/wp-content/uploads/2022/04/vignette_SimpleFlow.nb_.html).

#### Gate-Based Flow Cytometry Analysis

We use gating to define cells and subpopulations of interest within the total flow cytometry sample. Although we have tried clustering algorithms to automatically draw gates we have found manual gating to be more accurate and straightforward. Before gating, we transform the data in such a way that helps further distinguish subpopulations. We use a combination of logarithmic transformations for forward scatter (FSC) and side scatter (SSC) values and a logicle transformation for fluorescent values (Fig. 1B) before plotting and gating cells. We perform hierarchical gating to first define the cell population, distinguish individual cells from doublets, and then define cells with zero, one, and two or more copies of the reporter, using the zero-, one-, and two-copy control strains as guides (Fig. 1B). First, we gate for cells, by graphing forward scatter area (FSC-A) against side scatter area (SSC-A). This filters out any cellular debris (Fig. 1B, left panel). Second, we gate for single-cells by graphing forward scatter area (FSC-A) against forward scatter height (FSC-H) and draw a gate along the resulting diagonal (Fig. 1B, middle panel). Finally, we draw non-overlapping but adjacent gates to define three CNV subpopulations: zero-, one-, and two or more copies (Fig. 1B, right panel). To do this, we graph forward scatter area (FSC-A) against the fluorescent channel (Fig. 1B, right panel). In our case, we use the B2 channel area (B2-A) of a Cytek Aurora flow cytometer which detects GFP fluorescence with an excitation = 516 λ and emission = 529 λ.

The set of drawn gates defines the gating template which is then applied to sample data. Because of day-to-day variation in machine sensitivity, we have found that in some cases a universal gating template is not appropriate for a months-long evolution experiment. In this case, a gating template can be constructed for every timepoint using the corresponding measurement for the control populations at the same time point. Importantly, to assess the gates, we use a threshold of > 85% of control cells across all time points lying within the corresponding control gate. Gates are manually redrawn until it passes this assessment. The multiple attempts at drawing gates that fulfill these criteria is known in our lab as the “art of gating.” Once gates have been defined, we obtain the proportion of cells within each gate per time point and graph the proportion of each population with CNVs over time. These data are then used to quantify CNV dynamics as described below.

#### Quantifying Dynamics of CNVs

Using the proportion of the population containing a CNV per time point at the locus of interest, we calculate statistics, modified from (Lang et al. 2011), to summarize CNV dynamics. We calculate T_up_, the generation at which CNVs first appear, for each of the evolved populations. Previously, to calculate T_up_, we first defined a false positive rate of CNVs by calculating the proportion of one-copy controls appearing in the CNV gate across all generations (Lauer et al 2018). Then, we defined a threshold as the false positive rate plus one standard deviation. The generation at which the proportion of CNV-containing cells surpass this threshold is T_up_.

Alternatively, T_up_ can be defined as the time at which we first observe the proportion of CNV-containing cells surpass some threshold (e.g., 5%) for three consecutive generations. Our lab currently uses this second approach for calculating T_up_.

Next, we calculate S_up_, the percent increase in CNVs per generation for each evolved population. To do this, we take the natural log of the proportion of population containing CNVs divided by the proportion of population without CNVs for each time point. We plot these values across time and perform linear regression during the initial population expansion of CNVs. The slope of the linear fit is S_up_.

If CNVs are lost in the population, we calculate S_down_, the percent of CNV decrease, using the same approach as in S_up_, except the resulting slope is negative. If observed, we calculate T_down_, which is the generation CNVs go below the same threshold used for defining T_up_ (Lang et al. 2011)**.** In practice this quantity is not determined as we have not observed extensive loss of CNVs in our evolution experiments thus far.

CNV reporters provide insight into the dynamics of CNVs in adaptive evolution, facilitating further analysis. For example, we have used these data to estimate rates of CNV formation and fitness effects using neural network simulation-based inference (nnSBI) (Avecilla et al. 2022).

#### Non-gate-Based Flow Analysis Based on Fluorescence Signals to Visualize Copy Number Dynamics

In addition to gate-based flow cytometry analysis, we use the cell size (FSC-A) normalized fluorescent signal for additional analyses. For example, density plots of the normalized fluorescence are useful to examine as is the median normalized fluorescence for each sample. It is important to note that the median fluorescence convolutes CNV population frequency and variable copy number within cells and thus must be interpreted with caution. However, we have found that this metric provides a useful summary of population dynamics for comparison among replicate populations.

#### Isolation of CNV Lineages

The ability to detect individual cells with CNVs enables the isolation of CNV-containing lineages for subsequent analysis. We have successfully used FACS to isolate CNV-containing lineages by first sorting the subpopulation of cells with increased fluorescence indicating increased copy number, and then isolating clones through single colony purification. It is our standard practice to then use flow cytometry to confirm that the clonal isolate exhibits homogeneous fluorescence consistent with a CNV. Isolated CNV lineages can then be studied using fitness and phenotypic assays, and genome characterization by methods such as long-read nanopore sequencing, as described below.

#### Barcode Lineage Tracking Can Reveal Intra-population CNV Dynamics

The combination of a CNV reporter and lineage-tracking barcode enables additional levels of resolution on CNV-mediated adaptive evolution. Barcode sequencing allows the tracking of evolving lineages within populations by engineering unique genomic barcodes at a neutral locus in the background of a CNV reporter strain. Consideration of barcode length and estimated population size are important factors to consider when doing so (Johnson et al. 2023). The isogenic population of cells that vary only at the barcode site is experimentally evolved in a selective condition of interest. We then perform FACS-based sorting of the CNV subpopulation. The relative abundance of lineages over time can be estimated using DNA sequencing of the barcode sequence (Levy et al. 2015; Lauer et al. 2018; Nguyen Ba et al. 2019). Using this method, we have shown that CNV-lineage diversity is initially very large but decreases rapidly over time (Lauer et al. 2018). We note that in our lab, we identified a fitness defect in specific conditions due to the original location of the barcode landing pad (Levy et al. 2015; Lauer et al. 2018; Nguyen Ba et al. 2019) and thus re-engineered it at the *HO* locus in our strains using a single construct that combines both parts of the landing pad.

### Limitations

Despite the broad utility of CNV reporters, there are some inherent limitations and remaining challenges. We have observed non-linear scaling of GFP fluorescence in our zero-, one-, and two-copy controls. The median fluorescence of the two-copy control is less than two times that of the one-copy control. Although the median cell-size-normalized GFP fluorescence of controls are distinct, the distributions of our one-copy and two-copy control strains can display some degree of overlap (~ 6% on average) (Lauer et al. 2018). This overlap appears to increase between strains with two copies and three copies of the reporter, suggesting that there is diminishing resolution with higher copy numbers.

The CNV reporter system reveals population-level CNV dynamics and copy number but it is not informative about the CNV structures that are selected without subsequent analyses. Studies in our lab have shown that the suite of CNV-containing alleles identified using a CNV reporter consists of tandem duplications, large CNVs, complex triplications and inversions, translocations, and aneuploidy. However, the reporter does not distinguish between these different classes of CNVs, which must be analyzed using DNA sequencing.

### Future Directions

In our hands we have found that CNV reporters are a powerful tool to detect CNVs in real-time during experimental evolution with high resolution and repeatability. Future directions that we are currently exploring include using multi-color reporters at multiple loci and extending CNV reporters to diploid genomes and other microbial species. CNV reporters also enable larger population dynamics to be repeatedly evaluated without strain selection and sequencing, enabling CNV evolution experiments at scales comparable to classic long-term evolution experiments (Lenski 2023). In addition, we are exploring the application of machine-learning algorithms for enhancing the accuracy of CNV detection from flow cytometry data.

## Long-Read DNA Nanopore Sequencing

### Introduction

Once clonal lineages containing CNVs are isolated we use molecular approaches to resolve the genomic changes. Whereas short-read sequencing is widespread, well-established, and inexpensive, long-read sequencing offers numerous advantages for the identification of CNVs. Long-read sequencing, or third generation DNA sequencing, refers to a disparate group of technologies, such as SMRT, HiFi (Pacific-Biosciences) and Nanopore (Oxford Nanopore Technologies) sequencing (Logsdon et al. 2021). While molecular identification of CNVs preceded next-generation sequencing (NGS) technology (Jacobs 1981; Freeman et al. 2006), (Alkan et al. 2011) the predominance of NGS in the last decade has helped define our understanding of these phenomena (Pirooznia et al. 2015; Mahmoud et al. 2019). However, intrinsic limitations to NGS make it difficult to use in accurate characterization of CNVs, a task to which long-read sequencing is better suited (Ho et al. 2019; Mahmoud et al. 2019; Lavrichenko et al. 2021).

**Fig. 2:**
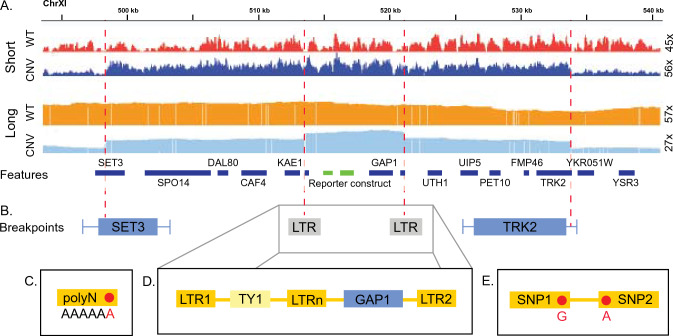
Advantages of long-read sequencing for resolution of complex or problematic genomic regions. Schematic shows read depth plots of real data (Spealman et al. 2022) visualized using IGV (Thorvaldsdóttir et al. 2012), here the horizontal shows Chromosome XI in *S. cerevisiae* and the vertical is the read depth at each location. Two strains are plotted; the ancestor strain lacking a CNV (WT, Red tracks, DGY1657) and an evolved strain with 4 copies of GAP1 (CNV, Blue tracks, DGY1740). Each strain has been sequenced using short-reads (SR) and long-reads (LR), with median relative depth shown at the right of each track. **A** SR depths are highly variable in both the WT and CNV strains, obscuring the resolution of the breakpoints (red dashed vertical lines), conversely the depth resolution using LR is far less variable and the breakpoints much easier to identify. **B** While SR can identify breakpoints within high-information regions such as CDS (SET3) or UTRs (TRK2) it fails to resolve breakpoints within low-information regions such as long-terminal repeats (LTRs). **C** LR offers advantages in some cases of poly-nucleotide resolution. **D** Resolving complex structures, such as a transposon sequence (TY1) mediated amplifications inside multimapping LTRs. **E** LR can also assist in determining phasing, that is, if two variants are physically contiguous or, instead, occur on distinct chromosomes or extra-chromosomal segments (Color figure online)

CNVs result in genomic rearrangements that are not present in a reference genome. These rearrangements necessarily include breakpoints that define the boundaries of the variant. When breakpoints occur in information rich sequences (e.g., complex and unique) they can produce short, novel sequences, which are identifiable using short-read data (Fig. 2). However, identification is much more difficult when breakpoints occur within information poor sequences (Nurk et al. 2022). Long-read sequencing can aid in the identification of breakpoints originating from regions with low-complexity (Fig. 2C), multimapping regions (Fig. 2D), or sequences with poor representation because of bias in the methodology itself (Kieleczawa 2006; Nurk et al. 2022). Long-read sequencing can also help in ‘phasing’, that is determining if two variants are physically contiguous with each other (Fig. 2E). By increasing the read size by several orders of magnitude, long-read sequencing enables low-information regions to be crossed allowing for the identification of breakpoints using distal markers (Mahmoud et al. 2019; Ho et al. 2019; Amarasinghe et al. 2020). Although long-read sequencing is a powerful methodology for the identification and characterization of CNVs, it is not without its own biases and limitations. We outline some of the approaches we take to mitigate these effects.

### Clonal Isolation and Experimental Methods

A difficulty in any evolution experiment is to meaningfully capture the diversity present within an actively evolving population. While sequencing the entire population in bulk is possible, important information (such as the co-occurrence of variants or phasing (Feng et al. 2021) is lost because of the discontinuity of the fragments (De Coster et al. 2021). Even in the ideal long-read sequencing scenario in which entire chromosomes were sequenced, there would still be discontinuity between chromosomes, confounding a complete picture of the evolving population of individuals (De Coster et al. 2021).

Traditionally, this problem has been solved by isolating clones from within a population, growing them rapidly to an abundance from which sufficient DNA can be isolated, and then constructing a library from those representative individuals (Schwartz and Sherlock 2016). Using the fluorescent CNV reporter aids in the accurate and rapid selection of clones of interest (Lauer et al. 2018).

Before clonal isolation, care should be made in the handling of evolved populations once the experimental evolution is completed. Critical aspects of experimental evolution such as genetic diversity and variant frequency can be rapidly distorted by exposure to alternative environments such as freeze–thaw (Sleight and Lenski 2007; Wing et al. 2020) or non-selective environmental conditions (Dragosits and Mattanovich 2013; Knöppel et al. 2018).

Even after isolation, care should be taken to prevent subsequent mutations arising from adaptation to other selective pressures or reversion of CNVs. Importantly, reversion rates are best known from phenotypic assays and the rates themselves vary depending on the mutation, the phenotype, the environment, and the selective pressure (Cairns and Foster 1991; Cairns et al. 1988). CNV reversions are less well characterized than SNVs and even then mostly in the context of extrachromosomal circular DNA amplification (Mishra and Whetstine 2016) and aneuploidy (Gorter de Vries et al. 2017; Gilchrist and Stelkens 2019).

In our lab’s experience using *S. cerevisiae* these issues can be avoided by keeping the generation count after removal from the evolution experiment as low as possible, avoiding unnecessary refrigeration and freeze thaw cycles. Our standard procedure when recovering from glycerol stocks, is to first plate on rich media (2–5 days, 30 °C) and when the colonies are large enough (~ 0.1 cm) inoculate overnight cultures (5 mL YPD, rotator drum, 30 °C) and harvest cells 12–24 h later.

Finally, caution should be exercised when drawing inferences from the clones selected about the state of the evolved population. While it is more likely that the clones recovered from a population represent the more abundant lineages within that population, it is not necessarily the case. Estimating population variant frequencies is difficult to do using NGS data because of the variability in site coverage and the difficulty in extrapolating from proportion of reads with variants to proportion of population with variants (Gautier et al. 2013). Given the reduced variance in coverage, variant frequencies from long-read sequencing can be more accurate, yet higher error rates, and sequencing depth limitations prevent it from being applied to low frequency variants (Schneider et al. 2022).

### Bioinformatic Approach

#### Base-Calling

ONT’s nanopore sequencing generates a signal that must be converted into DNA base calls. Given the complexity and computational requirements for transforming Nanopore’s raw signal to called bases, several solutions exist that are tailored for usage. These come, broadly, in two groups: those that are accurate but computationally expensive, such as Guppy (Wick et al. 2019), which supports three basecaller modes—Fast, High Accuracy, and Super-accuracy—with higher accuracy incurring a higher computational cost. These are contrasted with ‘ultra-lite’ base-callers that have magnitudes faster performance with a trade-off of lower accuracy, such as DeepNano-Blitz (Boža et al. 2020). Because of their lower accuracy these basecallers are used for preliminary base-calling, such as is required by real-time applications including field work or adaptive sampling. For our purposes, even when we use ultra-lite base-callers we save the FASTQ file for a second, offline, re-analysis using a slower but higher accuracy mode.

#### Sequence Alignment

Once a read has been base-called it can be aligned against a reference genome. A suite of read aligners specializing in handling long-read sizes and higher error rates exist (De Coster et al. 2019), with the most common being NGMLR (Sedlazeck et al. 2018), Minimap2 (Li 2021), and the recently released lra (Ren and Chaisson 2021). Sequence alignment is a common prerequisite for single-nucleotide variant calling where the reference genome can act as a contrast to the observed bases and structural variant calling where a re-arrangement of the genome will be identifiable as ‘split-reads’ when sequenced. However, because each aligner performs differently in their scoring and annotation of split-reads, often markedly so (Bolognini and Magi 2021, specifically Supplemental Fig. 7), it is recommended to try more than one during initial stages of analysis. In our lab we routinely use Minimap2.

#### Structural Variant Calling

A key feature of long-read sequencing is the ability to readily identify structural variants. Numerous tools have been developed that seek to perform that task. Importantly, benchmarking studies have shown that differences in sequencing technology (e.g., Nanopore or PacBio), aligners, and variant callers can generate significantly different sets of candidate CNVs (Mahmoud et al. 2019; Luan et al. 2020; Bolognini and Magi 2021). Caution should be exercised when interpreting identified CNVs and manual analysis, if possible, is highly recommended (Yang 2020). Notable variant callers are PBHoney (English et al. 2014) originally designed for PacBio reads but which performs well with Nanopore (Mahmoud et al. 2019; Luan et al. 2020; Bolognini and Magi 2021), CuteSV (Jiang et al. 2020) and Sniffles2 (Smolka et al. 2022) a major update to the popular Sniffles tool (Sedlazeck et al. 2018).

#### Single-Nucleotide Variant Calling

While nucleotide level accuracy for Nanopore is improving it has historically lagged behind that of Illumina short-read or PacBio HiFi (Sereika et al. 2022). However, recent improvements in Nanopore sequencing accuracy have made it possible to meaningfully identify single-nucleotide variants at moderate sequencing depths (Wang et al. 2021b) and continuing improvements in chemistry, such as the recently released Kit 14, hold even greater promise. Because long-read sequencing outperforms short-read sequencing in resolving challenging sequences and phasing (Wagner et al. 2022) and PacBio HiFi has very high accuracy, it does make long-read sequencing a compelling tool for SNV detection. Several options exist ranging from Clair3 (Zheng et al. 2021) a continuation of the popular Clairvoyant tool (Luo et al. 2020) to NanoCaller (Ahsan et al. 2021). A recent comparison found similar performance metrics between the most popular packages (Helal et al. 2022).

#### Sequence Assembly

An alternative to alignment is the de novo assembly of sequence reads, using tools such as Flye (Kolmogorov et al. 2019), Canu (Koren et al. 2017), or Raven (Vaser and Šikić 2021). Long-read assemblers aim to generate the longest and most accurate genomic reconstructions possible by joining reads together into contigs (Latorre-Pérez et al. 2020) and often perform well given sufficient depth (Zhang et al. 2022). In addition to long-read only assemblers, hybrid assemblers also exist that leverage short-read sequencing technology to aid in resolution and reduce artifactual calls (Brown et al. 2021). While most obviously useful for low-complexity metagenomics (Somerville et al. 2019; De Coster et al. 2021), genomes with complex SVs (Spealman et al. 2022) or de novo genome drafting (Wang et al. 2021a), assemblers are also useful for the identification of potential contaminants such as unintentional organisms, plasmids, and viruses. In our lab we have used both long-read and hybrid assemblers, metaFlye (Kolmogorov et al. 2020) and MaSuRCA (Zimin et al. 2013). Caution should be taken however, as no automated assembly is perfect, and when a comparable reference genome is available additional steps should be performed to validate the assembly, such as a whole genome alignment using dotplots, assembly correction (Alonge et al. 2019), and annotation (Shumate and Salzberg 2020).

### Limitations

While long-read sequencing has numerous advantages over short-read sequencing, especially in the identification of CNVs, it also has drawbacks and limitations that should be considered before adoption. Compared to Illumina or PacBio, Nanopore is relatively inexpensive upfront cost, as ONT’s starter kit of a flow cell, library preparation kit, and MinIon device is currently ~ $1000 USD, making it far cheaper than either competitor's smallest platform (Tvedte et al. 2021). However, after initial investment, PacBio’s Sequel II is cheaper per-base of sequence and delivers 2–5 times the data (Tvedte et al. 2021) often with higher levels of CNV resolution and accuracy (Mahmoud et al. 2021).

It is also worth noting that different experiments will be better suited to different technologies. Nanopore is best suited for smaller runs, such as whole genome sequencing of a single clonal yeast strain, or a dozen such strains with the addition of multiplex barcoding. Similarly, Nanopore is well-suited for the metagenomic analysis of low-complexity populations (Somerville et al. 2019). For larger genomes or communities of greater diversity, the efficiencies of scale shift in favor of PacBio (Tvedte et al. 2021; De Coster et al. 2021).

Finally, while long-read sequencing offers the ability to sequence across challenging sequences, the technologies have not completely overcome the biases and limitations of short-read sequencing (Amarasinghe et al. 2020). Nanopore, because of its reliance on pore conformation shifts and translocation rates, has difficulty with homopolymers (Amarasinghe et al. 2020), GC bias (Delahaye and Nicolas 2021), and large-scale secondary structures (Spealman et al. 2020) such as those generated by Origin Dependant Inverted Repeat Amplification (ODIRA) CNVs (Lauer et al. 2018). Furthermore, while long-read and ultra-long-read lengths can aid in the algorithmic determination of genome structures and structural variants, the implementation of these algorithms is still developing and will require additional manual refinement for the foreseeable future.

### Future Directions

#### Alternatives to Clonal Isolation

Whereas clonal isolation remains the simplest means of probing population CNV diversity, alternative methods are being actively developed. Single-cell sequencing methods are one appealing approach to increasing sample size and throughput. However, with the suspension of the 10× Genomics Single-Cell CNV technology in 2020 and Nanopore-enabled single-cell sequencing requiring additional hardware (Tian et al. 2021), single-cell approaches are currently outside of the reach of most labs.

#### Adaptive Sampling for Focused Analysis

Adaptive sampling is another method that has garnered attention for probing of diversity within populations (Loose et al. 2016; Miller et al. 2021; Mariya et al. 2022; Martin et al. 2022). Briefly, adaptive sampling uses Nanopore’s real-time sequencing in conjunction with ‘Read Until’ tools to evaluate the raw signal or base-called sequence and then perform some logical operation based on user defined criteria to decide if the read should continue to be sequenced or be rejected. It can also aid in the reduction of wasted sequencing as a saturation cut-off can be set that excludes sequences once a certain abundance has been reached (Payne et al. 2021). Unfortunately, the ONT supported Nanopore Adaptive Sampling (NAS) software is computationally intensive (Masutani and Morishita 2019) and requires hardware that can be prohibitively expensive. While free, computationally cheap, open source alternatives to NAS have been developed (Edwards et al. 2019; Payne et al. 2021; Ulrich et al. 2022) they are often rapidly rendered obsolete by ONT’s software update regimen, updates often lack backwards compatibility and often feature the removal of previous versions from online repositories. Despite these challenges, adaptive sampling is still an active area of research undergoing rapid development that may present an attractive approach in the near future.

#### Deep Signal Identification

With the advent of big data and accessible machine-learning (ML) and deep-neural network (DNN) platforms a trend has begun to emerge that seeks to extend beyond sequence level information to deeper biological signals that are present within the data but which are subtle, complex, or poorly understood (Wan et al. 2022). These tools can already identify the differences between nuclear and mitochondrial genomes (Danilevsky et al. 2022) as well as human and bacterial genomes (Bao et al. 2021). Species-specific basecallers trained on the genomes of specific species of plants are able to outperform ‘universal’ base-callers (Ferguson et al. 2022). Similarly, a rise in species-specific variant callers such as PEPPER-DeepVariant (Shafin et al. 2021) suggest information rich differences exist in variants as well. Currently, it is unclear what aspects of genome and variant biology are causing the improved performance but one possibility is altered patterns of DNA modifications such as methylation and chromatin accessibility (Wan et al. 2022). While these are known to change under aneuploidy in humans (Veronese 2020) and yeast (Mulla et al. 2017) it is not known if this would also extend, in whole or in part, to CNVs. However, the capacity to directly detect CNVs, SVs, and supernumerary chromosomes regardless of the underlying sequence would present a powerful tool for understanding genome dynamics and evolution.

## Conclusion

CNVs are an important class of genetic variation with important roles in adaptive evolution. Experimental evolution is well-suited to studying the role of CNVs and addressing fundamental questions about CNV diversity and dynamics, molecular processes that promote or inhibit the formation of CNVs, and the consequences of CNVs for the organism. Here we have summarized two tools that are critical for the efficient and accurate study of CNVs. The combination of CNV reporters and long-read sequencing enables unprecedented resolution of CNV-mediated adaptation and opens the door to a range of newly addressable questions.
